# Custodiol - N versus Custodiol: a prospective randomized double blind multicenter phase III Trial

**DOI:** 10.1186/1749-8090-10-S1-A73

**Published:** 2015-12-16

**Authors:** Gábor Szabó, Alexander Weymann, Bastian Schmack, Dominika Badowsky-Zyla, Gábor Gábor Veres, Florian Wagner, Ardawan Rastan, Thorsten Doenst, Matthias Karck

**Affiliations:** 1 University of Heidelberg, Heidelberg, 69120, Germany; 2 University of Hamburg, Hamburg, 20251, Germany; 3 Heart Center Rotenburg, Rotenburg, 36199, Germany; 4 University of Jena, Jena, 07747, Germany

## Background/Introduction

HTK-Solution (Custodiol) is a well-established cardioplegic and organ preservation solution. We currently developed a novel HTK based solution Custodiol-N which includes iron chelators to reduce oxidative injury as well as L-arginine, to improve endothelial function.

## Aims/Objectives

In the present first-in-human study, Custodiol-N was compared with Custodiol in patients undergoing elective coronary bypass surgery.

## Method

The study was designed as prospective randomized double blind non-inferiority trial. Primary end-point was area under the curve (AUC) of creatine kinase MB (CKMB) within the first 24 hours after surgery. Secondary endpoints included, peak CKMB and troponin-T and AUC of troponin-T release, cardiac index, cumulative catecholamine dose, ICU-stay and mortality. All values are given as mean ± SD, p < 0.05 was considered as statistically significant.

## Results

Early termination of the trial was performed per protocol as the primary non-inferiority end point was reached after inclusion of 101 patients. Patient characteristics, medical history, operation and cross-clamp times did not differ between the groups. CKMB AUC (878 ± 549 vs. 778 ± 439 h*U/l, non-inferiority p < 0.001) and Troponin-T AUC (12990 ± 8347 vs. 13498 ± 6513 h*pg/ml, non-inferiority p < 0.001) was similar in both groups. Although the trial was designed for non-inferiority, peak CKMB (52 ± 40 vs. 41 ± 30 U/l, superiority p < 0.002) was significantly lower in the Custodiol-N group. Cardiac index, catecholamines ICU-stay and mortality (1 death in the control group) was similar in both groups.

## Discussion/Conclusion

This study shows that Custodiol-N is safe and provides similar cardiac protection as the established HTK-Custodiol solution. The significantly reduced peak CKMB levels in the Custodiol-N group may implicate a beneficial effect on ischemia/reperfusion injury in the setting of coronary bypass surgery.

